# Suicide by Proxy: Murder as a Displaced Form of Suicidal Derive: A Case Series

**DOI:** 10.1192/bjo.2026.11789

**Published:** 2026-06-30

**Authors:** Yasir Karkosh, Bader al Habsi, Salim Alhuseini

**Affiliations:** MOH, Muscat, Oman

## Abstract

**Aims:**

Suicidal and homicidal behaviors are typically assessed as distinct risk domains, despite overlapping psychopathology in psychosis and severe mood disorder. We introduce the new concept Suicide by Proxy as a psychodynamic formulation in which homicide (or attempted homicide) represents a displaced enactment of suicidal intent when direct self-destruction is inhibited. Drawing on Freudian models of internalized aggression, persecutory superego dynamics, and object-related conflict (Oedipal/Electra configurations), we aim to illustrate how self-directed destructive drive may be externalized toward a symbolic other.

**Methods:**

Three retrospective forensic psychiatric case reports were analyzed using a psychodynamic framework. All cases involved severe mental disorder (schizophrenia-spectrum or major depressive episode with psychotic features), documented suicidal ideation and/or attempts, and a homicide or attempted homicide. Data sources included clinical interviews, collateral histories, medical/legal records, and psychological testing (including MMPI and SIMS). Identifying details were removed or altered to preserve confidentiality without changing clinical meaning.

**Results:**

Case 1:A 26-year-old man with paranoid schizophrenia and persecutory delusions purchased a firearm intending suicide but reported inability to act. He subsequently killed an unknown man in a mosque, then left the weapon beside the body. The act is conceptualized as displacement of self-destruction onto an authority-linked symbolic target, enabling rebellion against a persecutory superego without direct suicide or parricide.

Case 2:A 39-year-old woman with psychotic depression and intense suicidal communications stabbed her sleeping father after planning to kill herself in his presence, then attempted self-stabbing. This is formulated as a negative Electra configuration: ambivalent attachment and rage toward a passive betrayer father, with psychosis collapsing boundaries between self-attack and object-attack.

Case 3:A 36-year-old woman with schizophrenia, shaming maternal criticism, and relapsed hallucinations broke a mirror and attempted to attack her mother with glass while concealing suicidal intent. The mother is conceptualized as a proxy self-representing the rejected, humiliated part of the patient targeted for annihilation.

**Conclusion:**

Across cases, homicidal acts functioned as symbolic self-attacks emerging from internalized aggression, persecutory superego pressures, and unresolved parental-object conflict. Suicide by Proxy may help forensic clinicians integrate suicidality into violence formulations, prompting dual-screening for suicidal and displaced homicidal risk in severe mental disorder. Further empirical work is needed to test the model beyond case-series evidence.

